# Co-production and evaluation of an e-learning resource to improve African-Caribbean families’ knowledge about schizophrenia and engagement with services: a pilot randomised controlled trial protocol

**DOI:** 10.1186/s40814-018-0368-3

**Published:** 2018-11-20

**Authors:** Henna Lemetyinen, Juliana Onwumere, Richard James Drake, Kathryn Abel, Carol Haigh, Georgina Moulton, Dawn Edge

**Affiliations:** 1 Division of Psychology & Mental Health, Faculty of Biology, Medicine and Health, School of Health Sciences, The University of Manchester, Manchester, UK; 2 King’s College London, Department of Psychology, Institute of Psychiatry, Psychology & Neuroscience, London, UK; 3 South London and Maudsley NHS Foundation Trust, Bethlem Royal Hospital, Monks Orchard Road, Beckenham, Kent BR3 3BX UK; 4 Greater Manchester Mental Health NHS Foundation Trust, Bury New Road, Prestwich, Manchester, UK; 5 Manchester Metropolitan University, Brook Building, Bonsall Street, Manchester, UK; 6 Division of Informatics, Imaging and Data Sciences, Faculty of Biology, Medicine and Health, The University of Manchester, Manchester, UK

**Keywords:** African-Caribbean, Schizophrenia, Psychosis, E-learning, Digital health

## Abstract

**Background:**

A higher proportion of African-Caribbean people in the UK are diagnosed with schizophrenia spectrum disorders than other ethnic groups. High levels of shame and stigma at individual and community levels contribute to delayed access to care, potentially increasing the duration of untreated psychosis and so worsening outcomes. Inferior access, more coercive care, and worse outcomes have created a ‘circle of fear’ of mental health services within African-Caribbean communities. This further discourages early engagement with statutory services and increases the burden of care for families living with schizophrenia.

Providing tailored and relevant information about psychosis (psychoeducation) has the potential for improving outcomes for patients and families. However, there are no culturally appropriate psychoeducation programmes for African-Caribbeans in the UK. We aim to determine whether an e-learning resource, co-produced with African-Caribbean stakeholders to improve knowledge about psychoses, would be culturally acceptable and accessible to members of this population.

**Methods:**

A pilot randomised controlled trial of the feasibility of co-producing and testing a novel e-learning resource to improve knowledge about and attitudes towards schizophrenia in African-Caribbean families. We will seek to recruit 40 participants, aged ≥ 16 years, either to receive the intervention or as controls. They will self-refer or be referred via inpatient and wellbeing services, family and carers’ forums, statutory community mental health teams, and voluntary sector/non-governmental agencies (NGOs). Participants will complete the Ca-KAP, ASMI, and SF-12. Acceptability will be explored qualitatively via focus groups and individual semi-structured interviews.

**Discussion:**

The proposed trial will demonstrate the feasibility of conducting a fully powered RCT to evaluate the efficacy of an e-learning resource about schizophrenia with African-Caribbean families. Qualitative work will explore the intervention’s accessibility and barriers/facilitators to participation, including attitudes to randomisation. These data will facilitate further refinement of the intervention.

**Trial registration:**

ISRCTN11394005, retrospectively registered 20/03/2018.

## Introduction

African-Caribbeans living in the UK experience significantly higher rates of diagnosis with schizophrenia spectrum disorders [1] coupled with inequalities in accessing psychological interventions despite initiatives to address these issues [2–4]. ‘A circle of fear’ comprising delayed access to care, involuntary detention, coercive treatment, and poor outcomes has been repeatedly observed in African-Caribbean communities [5–7].

Delayed access to diagnosis and treatment can increase the duration of untreated illness, severity, and chronicity of symptoms, family tensions, and negative perceptions of the burden of care [8, 9]. Escalating family hostility, often resulting in police involvement, increases patients’ risk of estrangement from their families, social isolation, relapse, and rehospitalisation [5]. Evidence suggests that community-based education programmes could be effective in improving access and engagement in the African-Caribbean population [10, 11] who continue to be perceived by services as high risk and ‘hard-to-reach’ despite evidence to the contrary ([6]). Even upon accessing treatment, relatives and carers continue to feel disengaged from services [12], as psychoeducation is not routinely offered in UK community mental health services [13].

The UK’s National Institute for Health and Care Excellence (NICE) guidelines for schizophrenia management (2014) recommend the combination of medication with psychological therapy, such as family intervention [14]. The guidelines specifically recommend that psychoeducation is offered to carers of people diagnosed with schizophrenia [14]. Research shows that negative attitudes and stigma in the wider community, as well as within service users’ home environment, can negatively affect recovery [15], suggesting a need to make psychoeducation available to extended family and community members.

Previous studies of psychoeducation for schizophrenia and psychosis have been conducted with predominantly White European and American samples [16–22]. There have been some attempts to implement psychoeducation programmes in East Asian cultures with encouraging results. A Malaysian psychoeducation programme targeted caregivers of persons with schizophrenia and was reported to reduce the burden of care and improve caregivers’ knowledge of the illness, whilst improving relapse and hospital readmission rates for patients [23]. Several family psychoeducation trials from China have also reported improved outcomes for families, carers, and service users [24–26]. A psychoeducation programme delivered to Japanese families and caregivers improved their depressed mood, anxiety, and relationship with the service user [27]. However, there is little published evidence about the application of family educational interventions in ethnic minorities in the UK [14] taking account of local cultures and environments in which families live. Moreover, there are currently no evidence-based and/or user-informed culturally appropriate learning resources specifically aimed at African-Caribbean families with schizophrenia. This is a critical omission given the elevated risk within the UK’s African-Caribbean population [1, 28], inferior clinical outcomes, and poor service experience including lack of access to psychological care [4]. NICE Schizophrenia Guidance acknowledges deficiencies in the provision of family and culturally adapted resources and the need to work with African-Caribbean stakeholders to develop culturally-appropriate interventions [14].

A modern solution to improving access to health interventions is to make them scalable using the digital space. Health technology (i.e. e-health, m-health, tele-health), including websites, mobile applications (apps), and teleconferencing, is now acknowledged as a key element in NHS innovation, providing a wide range of information and support to individuals with mental illness and their families [29–31]. The potential for e-health to challenge inequalities in mental healthcare for marginalised populations lies in part in its ubiquity [32], enabling scalable, low cost, and timely access to information, support, and care across geographical areas through internet access [33].

## Aims of the trial

The aim of the study is to test the feasibility of implementing and evaluating a non-commercial e-learning resource among African-Caribbean families with schizophrenia family members in order to improve their knowledge and attitudes about the illness and about psychosis in general. The feasibility of recruitment, data collection, retention, and views on the intervention’s acceptability will inform a larger-scale RCT. This feasibility pilot has three primary outcomes:

i. Recruitment of relatives and carers of people with schizophrenia
ii. Participant retention
iii. Participant attrition

Secondary outcomes include:

iv. Improved knowledge about schizophrenia and psychosis
v. Improved attitudes about schizophrenia and psychosis
vi. Acceptability of the intervention

## Method

### Study design

The study is a parallel, two-arm feasibility RCT examining relatives’ and carers’ knowledge and attitudes about schizophrenia at three time-points: pre-intervention, post-intervention, and 3-month follow-up.

**Fig. 1:**
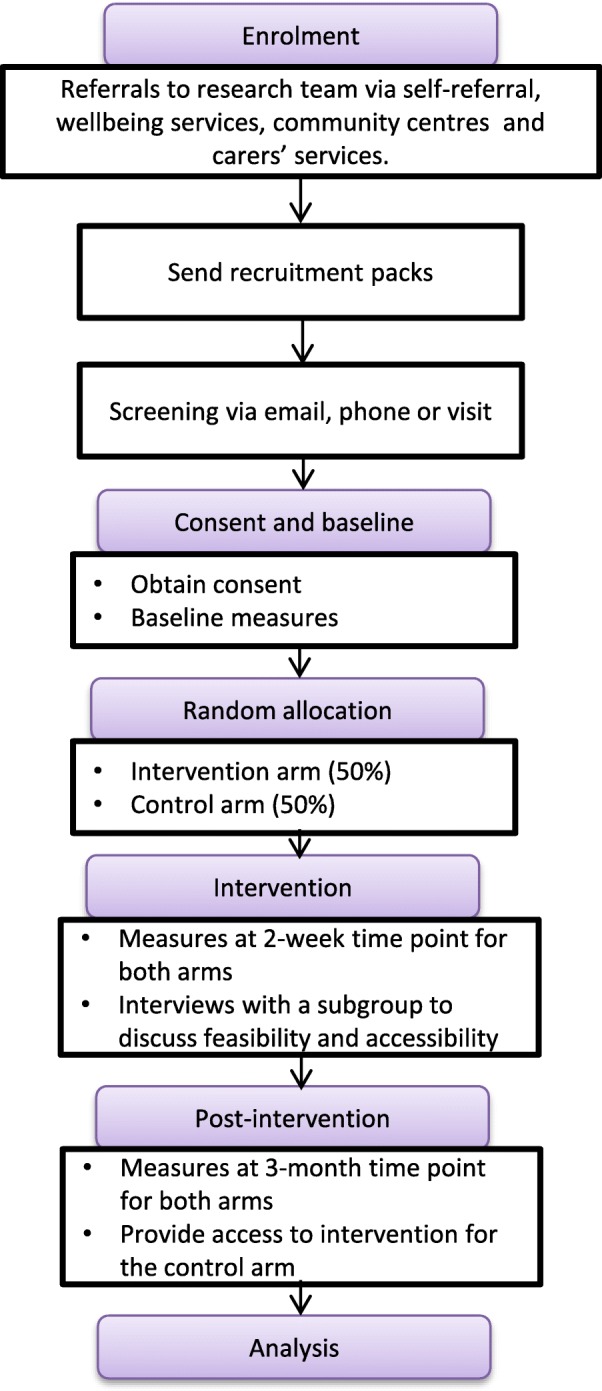
CONSORT diagram illustrating the pilot RCT procedure

The intervention will not be tested against a comparator. Instead, participants in the control arm will be administered the standardised measures without accessing a learning resource about schizophrenia. They will be provided access to the learning resource upon the completion of data collection (Fig. 1).

In addition, focus groups and one-to-one interviews will explore the feasibility and acceptability of the intervention. This qualitative work will afford opportunities for stakeholders to provide detailed feedback on the content, design, functionality, and cultural appropriateness of the intervention, which are unlikely to be captured by standardised measures.

### Participants and recruitment

We shall recruit 40 relatives and carers of African-Caribbean persons diagnosed with schizophrenia or other non-affective psychosis (DSM-V schizophrenia or ICD-10 F20-29: schizophreniform disorder, schizoaffective disorder, delusional disorder, or psychosis not otherwise specified). The sample size is based on previous community psychoeducation studies that report equivalent or smaller sample sizes as appropriate for assessing the feasibility of novel psychoeducation interventions [11, 34–36]. Recruitment will be facilitated by support from relevant organisations, such as the National Institute for Health Research (NIHR’s) Clinical Research Network (CRN), African and Caribbean Mental Health Services (a non-governmental organisation (NGO)), community centres, and patient and carer advocacy and support services. We shall raise awareness of the study in African-Caribbean communities via community engagement events, newspapers and radio, and social media.

### Inclusion criteria

i. Participants must be either related to and/or informally care for a person of African-Caribbean heritage with a diagnosis of schizophrenia spectrum disorders (F20-29). Participants are *not* required to be primary carers, as improving attitudes might yield positive gains even when accessed by wider community, e.g. extended family [15]
ii. Acknowledging ethnic diversity within families, participants need not be African-Caribbean themselves. For example, a White British parent of a child of Mixed White and African-Caribbean heritage is eligible to take part
iii. Minimum of 16 years of age prior to obtaining consent. There is no upper age limit
iv. Sufficient fluency in English to enable engagement with the intervention (currently only available in English) and completion of measures
v. Ability to provide written, informed consent

### Exclusion criteria

Individuals involved in contributing to the development of the resource (e.g. participated in consultation and feedback events on content and usability) will be excluded from the trial as such involvement might have increased their knowledge about schizophrenia and psychosis and/or generated more positive attitudes to mental illness and resource.

Persons under the age of 16 or persons who are not family members or informal carers will be excluded from the trial.

Persons diagnosed with schizophrenia spectrum disorders or other forms of psychoses will be excluded as the intervention is designed for families and carers versus service users.

### Intervention development

The intervention is a web-based e-learning resource to improve knowledge about schizophrenia and related psychoses among the families and carers of African-Caribbean service users with these diagnoses. Adopting a Community-Partnered Participatory Research (CPPR) approach [37, 38], resource development was informed by qualitative data collection via five focus groups with African-Caribbean stakeholders. Patient and public involvement (PPI) has been an increasing priority for health research in the UK. The government-funded National Institute for Health Research (NIHR) founded INVOLVE (http://invo.org.uk) to promote and support PPI in health research delivery. INVOLVE describes public involvement as “research activity conducted ‘with’ or ‘by’ members of the public, as opposed to ‘for’ or ‘about’ them”. The reasons for greater PPI involvement are manifold, including for example social justice and democratisation—those affected by research (the public) should have a right to provide input. Additionally, increasing research relevance and PPI improves research quality by providing involvement opportunities for members of the public as ‘experts by experience’ [39].

The aim of the focus groups was to inform the content, appearance, functionality, design, and delivery of the resource. This was achieved by facilitated discussions on the above topics using a semi-structured topic guide and related activities, such as watching short video clips, reading and discussing blog posts, and reviewing already available mental health resources. Focus groups comprised the following stakeholders:

- Persons with schizophrenia spectrum disorder diagnoses (*n* = 7)
- Relatives and carers (*n* = 6)
- Lay community members (*n* = 7)
- Young ($\overline{x}$ = 25 years) mixed group (consisting of relatives and community members) (*n* = 6)
- Design focus group (consisting of relatives, carers, and community members) (*n* = 8)

Stakeholders were recruited by advertising via community groups and institutions (such as Black Majority Churches, local charities, and other third sector organisations), public spaces (e.g. libraries and notice boards in local businesses), black and minority ethnicity (BME) networks, and local universities.

Our findings showed that the stakeholders prioritised information about symptoms and treatments. They also emphasised the importance of the resource being interactive to keep end-users engaged without compromising usability for any user groups, including older adults or those with lower IT literacy. Stakeholders emphasised the need for positive personal stories or case studies to counteract negative stereotypes of service users and families affected by schizophrenia and psychosis. In particular, the ‘young mixed group’ advocated for stories that would aim to illustrate schizophrenia and mental healthcare from a number of different perspectives, including those of the service user, various family members, and mental health professionals.

### E-learning resource

The e-learning resource, called *Culturally appropriate Schizophrenia Psychological Education Resource* (CaSPER), consists of thirteen fact-based topics on psychosis such as “*Schizophrenia and Black Caribbean people in the UK*”, “*Symptoms*”, “*Family and relationships*”, and “*Recovery and illness management*”. The topics originate from the focus groups and previous community work to identify African-Caribbean community members’ mental health needs and research priorities [38], which highlighted the need for more culturally appropriate information about schizophrenia.

CaSPER features nine fictitious stories, illustrating key elements of the factual information from key stakeholder perspectives, namely service users, family members, the police, and healthcare professionals. The stories feature an African-Caribbean family of three. As a young woman, ‘Jenny’ (who is married to ‘Michael’) was diagnosed with schizophrenia and admitted to hospital under a section of the Mental Health Act, a legislation that is effective in England, Wales, and Northern Ireland only (Scotland has its own devolved system). At the end of her story, ‘Jenny’ talks about her recovery journey, including how making changes in her life is helping her stay well. More recently, her 20-year-old son ‘Paul’ experienced a first episode of psychosis. His story outlines his care pathway via an Early Intervention Service (EIS). The aim of the story is to provide information about different parts of mainstream mental health services in clear, accessible formats as African-Caribbeans have indicated that lack of such information contributes to fear of services and delayed engagement [38]. The family’s story also illustrates different care pathways: Jenny’s coercive pathway, which typifies that of many African-Caribbean services users [40–42] contrasts with the more positive model of her son’s story of help-seeking and receiving care in the early stages of the illness.

Additionally, the resource includes an exhaustive list of local services for service user and carers and a glossary of key terminology as members of this community have indicated that access to this information might facilitate engagement, access, and advocacy [38]. The resource also contains short, informal multiple-choice quizzes on most topics to create an interactive e-learning environment.

In response to stakeholders involved in the co-production process, the resource will be available in both online and DVD formats to increase accessibility particularly among resource-users with limited IT access and/or low IT literacy. The DVD version is delivered as a ‘blended learning’ package with a booklet containing supporting information that cannot be delivered on a DVD, such as the multiple-choice quizzes. All factual content and the fictitious stories are available as audio recordings and as text to improve accessibility. The online version of the e-learning resource was designed to be accessible on a number of electronic devices, including a computer, a tablet, and/or a smartphone.

The intervention’s usability was informed by previous research, particularly that of Rotondi and colleagues (2005, 2010, 2017). They produced and tested a web-based e-learning schizophrenia learning resource to educate service users and their families. They identified a number of design features that improve website accessibility, such as flat design (everything being accessible via a few mouse clicks as possible), minimising display distractions (e.g. decorative images) and reading ease (using lay-friendly vocabulary and grammar). These features were incorporated into intervention development as they were congruent with focus group feedback.

### Outcomes and measures

The primary objectives will be:

- To test the feasibility of recruiting relatives and carers in the community and via relevant services, such as community mental health teams, home treatment teams, early intervention services, and support groups for carers
- To compare recruitment, uptake, and retention in both arms of the study
- To test and compare attrition in both arms of the study

We shall also collect demographic data, such as date of birth, country of birth, ethnicity, and employment status at baseline.

Secondary objectives will be to collect data on (i) improved knowledge of schizophrenia, (ii) improved attitude towards mental illness (iii) quality of life, and (iv) qualitative reporting of acceptability of the intervention.

They will be examined using the following outcome measures:

#### Culturally adapted Knowledge About Psychosis (Ca-KAP) questionnaire

Ca-KAP was developed specifically to assess knowledge and understanding of schizophrenia in the African-Caribbean population [43]. The Ca-KAP is based on a standardised instrument, Knowledge About Schizophrenia Interview (KASI) which has been shown to be successful at measuring families’ understanding of schizophrenia [44–46]. However, KASI was validated using a White British sample and has not been extensively used with African-Caribbean families. Additionally, the language was outmoded and the content did not readily allow for alternative models of mental illness. These were addressed during the cultural adaptation process. The resulting Ca-KAP consists of the following seven subscales: diagnosis, symptoms, and problems relating to psychosis, cause, medication, other treatments and services, course and outcome, and management. The themes of the Ca-KAP overlap with the content of the intervention (e.g. symptoms, medication, and treatments).

#### Attitudes to severe mental illness scale

Attitudes to Severe Mental Illness Scale (ASMI) is a validated assessment [47] which draws from a number of older standardised measures of attitudes to mental illness, such as the Opinion on Mental Illness (OMI) Scale and Community Attitudes to Mental Illness (CAMI). ASMI was selected by stakeholders in phase 2 focus groups as the most acceptable attitudes measure out of five assessments. The stakeholders liked the relatively short length of this 30-item questionnaire. They also found the wordings of the items accessible. ASMI consist of four subscales (stereotyping, optimism, coping, and understanding) comprising 30 statements about people with mental illness, such as “People with severe mental illness are failures”, “A person with severe mental illness can be trained in an occupation”, and “The friends should not abandon a person when he/she is suffering from severe mental illness”*.* Respondents are asked to rate their degree of agreement/disagreement with each statement on a Likert scale with scoring as follows: “agree” (4), “rather agree” (3), “rather disagree” (2), “disagree” (1), to “do not know” (0). Negatively worded items are reverse scored. A high total score reflects a positive attitude towards persons with mental illness.

#### SF-12 version 2

Adopted from SF-36 [48], SF-12 v2 is a practical, reliable, and valid self-reported measure of functional health 12 questions to and well-being. It is widely used to monitor population health, analyse disease burden, and predict costs and is particularly useful at the community level as it can be completed in 2–3 min. A preference-based utility index (SF-6D) has been developed from the SF-12 to facilitate economic evaluation and estimate quality-adjusted life years (QALYs). This will be important for future studies. However, in this study, we shall examine scores before and after and at the 3-month follow-up to provide preliminary data on whether using the learning resource has improved relatives’/carers’ health.

### Quantitative data collection

Participants accessing the intervention online will be provided a choice between completing electronic versions or hard copies of the measures. Participants using the DVD version will complete hard copies of the measures. The control group will complete the same measures at the same time points. However, they will access the intervention only after the collection of time point 3 outcome data. Participants will be informed of this procedure prior to collecting consent.

### Procedure

**Fig. 2:**
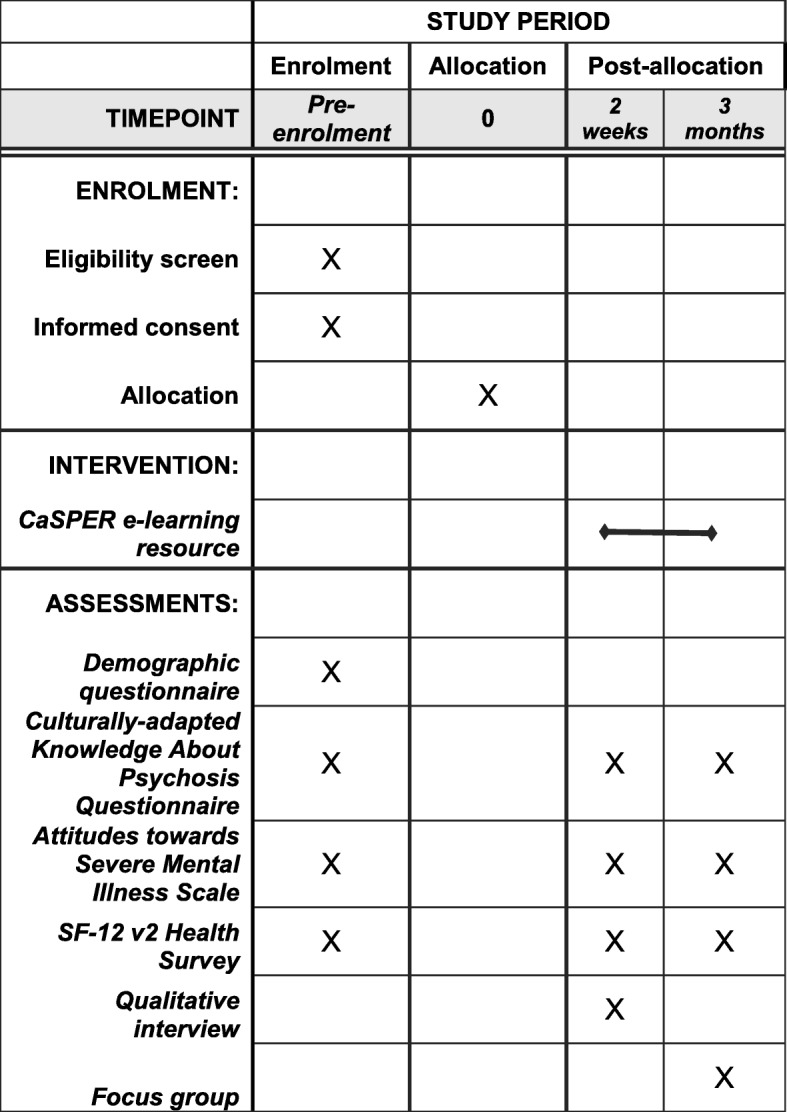
SPIRIT figure of the study procedures

Data will be collected in both intervention and control groups at three time points (see Fig. 2):

#### Pre-intervention(baseline assessment)

Basic demographic details and quantitative outcome measures will be completed either online, using a hard copy, or in a meeting with a research assistant. Participants in the intervention arm will select whether to access the resource via online e-learning or the DVD and booklet format.

#### Post-intervention

Within 2 weeks after completion, participants in both arms will be invited to complete the quantitative outcome measures. We shall also collect qualitative acceptability data using one-to-one semi-structured interviews with intervention group participants.

#### Three-month follow-up: outcome

There are two components to this phase. First, all participants will be tested on the outcome measures. Next, we shall collect qualitative feasibility data with 15 participants. A purposefully selected sample of participants from the intervention arm (*n* = 10) will explore in detail their views about the resource’s accessibility, perceived usefulness, and impact on attitudes and beliefs about schizophrenia. We shall also explore their views about the outcome measures, feasibility of data collection, and participation in the study more generally. Qualitative interviews with members of the control group (*n* = 5) will enable us to gather data on their perceptions and experience of recruitment and randomisation and their views on factors that influence recruitment and retention among control group members. A subsample of 15 participants was considered appropriate for this phase of the study to minimise participant burden (i.e. not inviting everyone to take part) but still allowing us to explore the feasibility of the pilot itself. We shall develop a sampling frame based on participants’ demographics to ensure a maximum variation sample in respect of key criteria such as age, prior knowledge of schizophrenia, and gender. A topic guide will be developed to facilitate this component of the study based on issues that emerge at time-points 1 and 2 and the available literature.

### Randomisation

Participants will be randomly assigned to either an intervention or control group of equal size (20 in each arm), using the web-based randomisation service (http://www.sealedenvelope.com). Group assignment will be recorded in a database, and the result of each randomisation emailed to the trial administrator.

Before consenting to participation in the study, respondents will be informed that there is a 50/50% probability of being assigned either to the intervention or control arms. This is to ensure that participants understand the study design and the fact that, although everyone will eventually be able to access the intervention, those in the control arm will not be able to do so straight away. Instead, control arm participants will be informed that they will be given access after completing a three-month follow-up.

### Statistical analysis plan

Demographic data will be analysed using basic descriptive statistics. Recruitment and retention rates into both arms will be calculated. We shall examine univariate associations of the outcome measures, using parametric or non-parametric tests as appropriate. We shall conduct exploratory tests of change during the course of the intervention using paired measures, such as the Wilcoxon paired-samples test or a *t* test, as appropriate according to the data.

### Qualitative data collection

Qualitative data on acceptability of the intervention will be collected via semi-structured interviews using a specially developed interview schedule (available on request). The schedule comprises 13 questions about participants’ experiences of using the intervention, covering usability, appearance, content, interactive features, and the extent the intervention meets the needs of African-Caribbean families affected by schizophrenia.

### Qualitative data analysis

Interviews will be digitally recorded with participants’ consent, transcribed with full anonymisation and checked for accuracy. Data will be explored using framework analysis [49]. Both a priori and emergent themes will also be included in the analysis. Framework analysis is particularly suitable for these data as it enables the analysis of responses to each question in the interview guide (a priori themes) with added flexibility of any arising (a posteriori) topics. NVivo (version 11) will be used to support data management and analysis. Coding and analysis will be lead and conducted by the Research Project Manager (RPM) with input from the senior author, a qualitative and mixed methods specialist.

### Data handling and record keeping

Participants will be able to choose to have their data collected online, on the phone, or by arranging a home visit with a researcher. Thus, we shall collect and store electronic and hard copy data. Electronic data will be securely captured and stored by Select Survey (selectsurvey.net) on a university-based server for confidential questionnaire data. Paper copies of data, including questionnaires and participant contact details, will be stored securely in a locked filing cabinet on university premises, in compliance with General Data Protection Regulation 2018. Electronic backup copies of data are password-protected. All team members accessing confidential data have completed Good Clinical Practice training.

## Discussion

This pilot trial is unique in evaluating the feasibility and acceptability of a novel, culturally-appropriate e-learning intervention to improve knowledge and attitudes about schizophrenia and psychosis in African-Caribbean families. According to the authors’ knowledge, it is also the only psychoeducation intervention for schizophrenia spectrum disorders that has been co-produced with African-Caribbean stakeholders. This pilot trial will examine the feasibility of recruiting and retaining participants from an ethnic group most likely to be diagnosed with schizophrenia spectrum disorders and among the least likely to receive psychoeducation. To inform further development of the intervention and a larger, fully-powered trial of its cost and clinical-effectiveness, we shall also assess the feasibility of collecting relevant outcome data. Importantly, for a community that regards itself as being ‘seldom heard’ versus ‘hard-to-reach’ [50] as commonly reported, we shall also evaluate African-Caribbean families’ perspectives on the intervention acceptability, accessibility, and utility.

### Potential strengths of the study

A significant strength of the study is that the development of the tested intervention was informed by key African-Caribbean stakeholders, including service users diagnosed with schizophrenia, their relatives and carers, and members of the wider community. This enabled the identification of relevant and culturally appropriate content such as acknowledging cultural differences in social norms. For example, ‘lack of eye contact’ is considered as a negative symptom of schizophrenia, whereas many African-Caribbeans would consider looking someone in the eye rude. This enhanced decision-making about the ‘look and feel’ of the intervention (its design and appearance) thereby increasing the likelihood of its acceptability to potential study participants. In addition to co-producing the intervention, stakeholders contributed to selecting the standardised outcome measures to ensure evaluation of issues that they considered most important such as improving attitudes to mental illness (the ASMI scale [47]).

Additionally, the accessibility of the intervention has been optimised by providing the content as text and audio recordings for end-users with either low literacy or for those who prefer listening instead of reading. To our knowledge, there are no other schizophrenia and psychosis information websites that offer users a choice between reading and listening to the same content. This is important to maximise the intervention’s accessibility and acceptability to most end-user groups, as highlighted by our phase 2 focus groups. We further increased accessibility by making the website compatible for access via computers, tablets, and smartphones, as well as by producing a DVD version for end-users without internet access.

### Potential challenges of the study

Based on previous research (Ref), we anticipate that participant recruitment will be challenging. Potential barriers to recruitment may arise from the fact that we are recruiting a sample consisting of a ‘minority within a minority’. This means that potential participants are members of not only an ethnic minority, but a minority of families affected by schizophrenia and psychosis who are also willing to engage in a trial, in contrast to previous reports of lack of recruitment of BME community members [51, 52]. Even though the incidence of schizophrenia and psychosis is elevated in the African-Caribbean community, evidence suggests that eligible participants, i.e. relatives and carers, are more likely to be ‘hidden’ in Black and Ethnic Minority communities [53, 54]. Furthermore, it is known that the uptake of NHS and social care-based service user and carer forums is low within this group. The difficulties services have engaging this group may limit the potential to recruit through them. Less is also known about what proportion of relatives and carers of persons with schizophrenia access support and wellbeing services within their communities.

The study design also has some limitations. The study in its current form is aimed at relatives and carers with family members diagnosed with schizophrenia/psychosis who have had some contact with mental health services. To address wider issues related to delayed access to care in the UK African-Caribbean population, the resource should be piloted with families who have not accessed services. This would provide insight into the intervention’s potential to impact help-seeking and care pathways in this group. We recommend future research to test CaSPER and other community-level, co-produced interventions with participants who do not yet have diagnoses, principally those experiencing first episode of psychosis and/or within Early Intervention Services.

## Abbreviations

ASMI: Attitudes to Severe Mental Illness Scale
BME: Black and minority ethnic
Ca-KAP: Culturally adapted Knowledge About Psychosis questionnaire
CAMI: Community Attitudes to Mental Illness Scale
CaSPER: Culturally appropriate Schizophrenia Psychological Education Resource
CONSORT: Consolidated Standards of Reporting Trials
CPPR: Community-Partnered Participatory Research
DSM: Diagnostic and Statistical Manual of Mental Disorders
EIS: Early Intervention Services
E-learning: Electronic learning
ICD: International Classification of Diseases
NGO: Non-governmental agency
NHS: National Health Service
NIHR: National Institute for Health Research
OMI: Opinion on Mental Illness Scale
PPI: Patient and Public Involvement
QALY: Quality-adjusted life years
RCT: Randomised controlled trial
RfPB: Research for Patient Benefit
SPIRIT: Standard Protocol Items: Recommendations for Interventional Trials
UK: United Kingdom
